# Hysterosalpingography: a potential alternative to laparoscopy in the evaluation of tubal obstruction in infertile patients?

**DOI:** 10.4314/ahs.v21i1.47

**Published:** 2021-03

**Authors:** Reyhan Gündüz, Elif Ağaçayak, Gülcan Okutucu, Özge Kömürcü Karuserci, Nurullah Peker, Mehmet Güli Çetinçakmak, Talip Gül

**Affiliations:** 1 Dicle University, Faculty of Medicine, Department of Obstetrics and Gynecology/Diyarbakır/Turkey; 2 Gaziantep University, Faculty of Medicine, Department of Obstetrics and Gynecology/Gaziantep/Turkey; 3 Dicle University, Faculty of Medicine, Department of Radıology/Diyarbakır/Turkey

**Keywords:** Infertility, hysterosalpingography, laparoscopy, tubal factor

## Abstract

**Background:**

Evaluation of the fallopian tubes are important for infertile patients. The two most important diagnostic procedures used to evaluate tubal patency are hysterosalpingography and laparoscopy.

**Objectives:**

To asses the hysterosalpingography and laparoscopy results of patients diagnosed with infertility and investigate the diagnostic value of hysterosalpingography in patients with tubal factor infertility.

**Methods:**

The hysterosalpingography and laparoscopy results of 208 patients who presented to the Obstetrics and Gynecology Clinic at Dicle University, Faculty of Medicine between January 2014- January 2018 were retrospectively evaluated. Hysterosalpingography and laparoscopy results were compared with regard to the investigation of the presence of tubal obstruction and of the pelvic structures that could cause tubal obstruction. The specificity, sensitivity, positive, and negative predictive values of hysterosalpingography were computed.

**Results:**

The number of patients evaluated was 208. The ratio of primary infertile patients was 57.2% and 42.8% was secondary infertile. Hysterosalpingography was found to have a specificity of 64.6%, the sensitivity of 81.3%, the positive predictive value of 56.4%, and a negative predictive value of 86% in the determination of tubal obstruction.

**Conclusion:**

Patients with suspected tubal infertility can primarily be examined using hysterosalpingography in consideration of the invasive nature and the higher complication rate of laparoscopy.

## Introduction

Infertility is defined as the failure of a couple to a clinical pregnancy after 12 months of regular sexual intercourse without contraception^1^. The most prevalent determinable female factors, which accounted for 81% of female infertility, were: ovulation disorder (25%), endometriosis (15%), pelvic adhesion (12%), tubal obstruction (11%), other tubal abnormalities (11%), and hyperprolactinemia (7%)^2^.

In hysterosalpingography (HSG), water or lipid-soluble contrast agent is used. HSG is the most commonly used diagnostic method to test tubal patency in patients suffering from infertility^3^. It is a standard diagnostic tool in the examination of tubal obstruction in all patients who are not planned to undergo laparoscopy (L/S) 4.

HSG also has therapeutic effects. In a systematic review of 12 randomized studies; subfertile women who underwent HSG with a lipid-soluble contrast agent demonstrated significantly higher rates of pregnancy than those who did not undergo HSG (odds ratio [OR] 3.30, 95% CI 2.00–5.43), and the use of lipid-soluble and water-soluble contrast agents were found to be comparable in terms of pregnancy rates (OR 1.21, 95%CI 0.95–1.54) 5.

L/S with chromopertubation is widely accepted as the “gold standard” method for evaluating tubal patency but L/S is an invasive and expensive method^6^. The results of L/S usually do not affect the initial treatment of the infertile couple when the infertility examination produces a normal result or indicates severe male factor infertility. Infertile women with a history of previous surgery or pelvic adhesion induced by pelvic infections or endometriosis can be examined using both L/S and chromotubation 7.

This study aims to determine the role and importance of HSG in the examination of female infertility by comparing its diagnostic value to the results of L/S in the evaluation of infertile women with a distal tubal factor. We aim to contribute to the literature by showing when and in which patients HSG can be used as a standalone alternative to L/S, which is an invasive method associated with high complication rates.

## Methods

This retrospectıve study included 208 infertile patients who presented to the Obstetrics and Gynecology Clinic in a university hospital between January 2014- January 2018, who underwent HSG and either showed pathologies suggesting bilateral/unilateral tubal factors or showed no pathology on HSG but underwent L/S for infertility that persisted for at least 6 months after the HSG examination. Approval was granted for this study by Dicle University Faculty of Medicine Ethics Committee (Date: 02.10.2019, Approval number: 199). Patients with uterine factors, male factors, smokers, premature ovarian failure, patients with chronic diseases, and history of abdominal surgery were excluded in the study. Patients with distal tubal obstructions on HSG and L/S were included in the study, proximal tubal obstructıons as it may be secondary to transient tubal spasms (20% of cases) or amorphous debris or minimal adhesions (40% of cases)^6^ were excluded in the study. Patients who were both primary and secondary infertile were included in the study. The demographic properties of the patients, duration of infertility, type of infertility, and menstrual regularity were recorded. An average adult menstrual cycle with a regular cycle of 28 to 35 days, a follicular phase of approximately 14–21 days, and a luteal phase of 14 days were considered as the reference^8^. The menstrual cycles of patients with 28 to 35-day cycles were regularly recorded. Patients with primary and secondary infertility were categorized into two groups. WHO defines primary infertility as the failure to conceive after one year of sexual intercourse without contraception and secondary infertility as the failure to conceive after the previous pregnancy^9^. In this study, we grouped primary and secondary infertile patients based on these definitions. Patient data were acquired from files in the hospital archives.

Women who failed to conceive after 12 months of regular sexual intercourse without contraception were considered infertile patients. In our study, when comparing HSG and L/S, L/S was used as a reference test. HSG was performed in the Radiology clinic of our hospital by a radiologist.

HSG was performed immediately after menstruation, in the follicular phase, without anesthetics, by administering a water-soluble contrast agent through the uterine cervix (ultravist 300, optiray 300), with scopy. The results of HSG were based on whether there was unilateral or bilateral distal tubal obstruction and results of patients' HSG with bilateral patent tubes were recorded as normal.

L/S was performed under general anesthesia, in the low lithotomy position, immediately after menstruation, in the follicular phase. Tubal patency was evaluated by administering methylene blue through the cervical os with the use of uterine manipulator during L/S. Laparoscopic findings were noted including tubal obstruction (right tubal obstruction, left tubal obstruction, bilateral tubal obstruction, unilateral or bilateral hydrosalpinx, and normal patients with two patent tubes), pelvic adhesion, endometriosis, myoma uteri, morgagni cysts, and diseases related to other pelvic pathologies. Pelvic adhesions detected on L/S were evaluated according to the American Fertility Association^10^ and pelvic adhesion was recorded as present or absent. Pelvic pathologies detected during L/S were noted according to the presence of a tubal obstruction in HSG and compared.

Statistical analyses were conducted using the SPSS 21 statistics software package. The quantitative data obtained in this study were presented in the form of arithmetic mean±standard deviation, and categorical data in the form of frequency (percentage). This study used descriptive statistics. The variables were analyzed using the Chi-square test. The specificity, sensitivity, and positive and negative predictive values of HSG were computed. Statistically, a p-value<0.05 was considered significant.

## Results

The number of patients who underwent HSG and L/S was 208. The ratio of the primary and secondary infertile patients were 119 (57.2%) and 89 (42.8%) respectively. The demographic properties of the patients are presented in Table-1.

**Table- 1:** Demographic properties of the patients (n=208)

n=208	Primary infertility (Mean ± SD)	Secondary infertility (Mean ± SD)
Age	28.9±5.5	31.8±5.9
Gravida	0.0±0.0	2.17±1.4
Parity	0.0 ±0.0	0.9±0.8
Abortus	0.0±0.0	1.1±1.5
Live	0.0±0.0	0.9±0.8
Duration of infertility	4.7±3.2	4.4±2.3

Data of the patients who und erwent L/S and HSG are presented in Table-2 with regard to the presence of tubal obstruction. Based on the results, 86 patients were found to have patent tubes on both HSG and L/S and 61 patients had unilateral or bilateral tubal obstruction on both HSG and L/S. Therefore, HSG and L/S results were compatible in 147 (70.6%) of the 208 patients whose tubes were found to be either patent or obstructed (p≤ 0.001). Meanwhile, 14 patients who had unilateral or bilateral tubal obstruction on L/S were found to have patent tubes on HSG, and 47 patients who had patent tubes on L/S were detected to have a unilateral or bilateral tubal obstruction in HSG (p≤ 0.001). In the present study, HSG was found to have a specificity of 64.6%, a sensitivity of 81.3%, a positive predictive value of 56.4%, and a negative predictive value of 86% in the detection of tubal obstruction (Table-3) 11.

**Table- 2:** L/S – HSG results with regard to tubal obstruction (n=208)

	L/S Tubes patent n(%)	L/S Unilateral or bilateral Tubes Obstructed n(%)	Total	Test; p
**HSG Tubes Patent**	86 (86)	14 (14)	100	χ^2^=40,642; p=0.000*
**HSG Unilateral or bilateral** **Tubes obstructed**	47 (43.5)	61 (56.5)	108	
**Total**	133	75	208	

* p≤0.001

Chi-square test

**Table 3:** HSG's sensitivity specificity calculation

Statistic	Value	95% CI
**Sensitivity**	81.33%	70.67% to 89.40%
**Specificity**	64.66%	55.91% to 72.75%
**Positive Likelihood Ratio**	2.30	1.78 to 2.97
**Negative Likelihood Ratio**	0.29	0.18 to 0.47
**Disease prevalence** (*)	36.06%	29.53% to 42.99%
**Positive Predictive Value** (*)	56.48%	50.16% to 62.60%
**Negative Predictive Value** (*)	86.00%	79.03% to 90.92%
**Accuracy** (*)	70.67%	63.98% to 76.77%

(*) These values are dependent on disease prevalence

L/S identified pelvic adhesion in 33 patients. Of the patients detected to have pelvic adhesion on L/S; 1 had myoma uteri, 3 had hydrosalpinx, 1 had an ovarian cyst, 1 had endometriosis, and 1 had a tubo-ovarian abscess. The remaining 26 patients did not demonstrate any pathologies that could explain pelvic adhesion. We detected in 22 (66.7%) of these 33 patients unilateral or bilateral tubal obstructions on the HSG examination. These patients were suspected of having the pelvic inflammatory disease.

Of the 108 patients whose HSG results indicated unilateral or bilateral tubal obstruction and who underwent L/S, 82 (62.6%) were detected to have pelvic pathologies visible on L/S (myoma uteri, hydrosalpinx, morgagni cyst, endometriosis, pelvic adhesion). Of the 100 patients with HSG results indicating patent tubes, 49 (37.4%) were detected to have pelvic pathologies on L/S (Table-4).

**Table-4:** L/S- HSG results with regard to pelvic pathologies

	L/S		Total	Test; p
	Pathology absent n (%)	Pathology present n(%)		
**HSG Tubes Patent**	51 (66.2)	49 (37.4)	100	χ^2^=16.146; p=0.000*
**HSG Tubes Obstructed**	26 (33.8)	82 (62.6)	108	
**Total**	77	131	208	

* p≤0.001

Chi-square test

Pelvic pathology: Myoma uteri, hydrosalpinx, morgagni cyst, endometriosis, pelvic adhesion

When the patients were evaluated with regard to the regularity of their menstrual cycles; there was no difference between patients with tubal obstruction and patients with patent tubes on HSG (p>0.05). However, patients detected to have pelvic pathologies on L/S presented significantly different menstrual cycles that were more irregular (p<0.05).

## Discussion

Infertility is a unique medical condition as it concerns not a single individual but a couple. HSG is a widely-used diagnostic method in the evaluation of tubal patency, diagnostic examination of infertility, and the detection of intrauterine anatomical defects. Our aim in conducting this study was to determine whether HSG was as successful as L/S in the detection of tubal pathologies. The results of our study suggest that HSG has a low error rate in patients with patent tubes, but L/S is warranted in patients with distal tubal pathologies. In the literature, Duraker and colleagues reported a mean age of 31.8 ± 5.7 and a mean duration of infertility of 30.7 ±3.7 months in infertile patients, with 64.7% primary infertile and 35.3% secondary infertile cases^12^. These results are consistent with the results of our study.

Another study that included 181 patients who underwent both L/S and HSG reported that both methods indicated normal tubes in 99 patients and obstructed tubes in 37 patients. Thus, HSG was a reference for L/S in 75% of the cases^13^. In the present study, 86 patients were found to have patent tubes on both HSG and L/S. On both HSG and L/S, 61 patients were found to have unilaterally or bilaterally obstructed tubes. Therefore, HSG was a reference for L/S in 147 (70.6%) of the 208 patients whose reports indicated either patent or obstructed tubes. Thus, our results are consistent with those of the cited study.

In a study conducted by Ngowa and colleagues, HSG was found to have the sensitivity of 86.6%, the specificity of 42.2%, the positive predictive value of 69.4%, and negative predictive value of 67.9% in the detection of distal tubal obstruction^14^. In the present study, HSG was found to have the sensitivity of 81.3%, the specificity of 64.6%, the positive predictive value of 56.4%, and negative predictive value of 86%. Thus, HSG could detect unilateral and bilateral tubal obstruction at a rate of 81.3%, however, the probability of the patients it diagnosed with tubal obstruction to have tubal obstruction was 56.4% and the probability of the patients it diagnosed with patent tubes to have patent tubes was 86%. The results of our study were consistent with the literature.

In a study conducted by Hassanein and colleagues, pathology was detected in L/S in 83% of 18 patients with normal HSG results. We found normal L/S results in 23% of 96 patients with abnormal HSG^15^. In our study, we detected pathology in 14% of 100 patients with normal HSG at L/S and L/S of 43.5% of 108 patients with HSG abnormal were evaluated as normal. The results of their study were not compatible with our study. While there were 114 patients in their study, there were 208 patients in our study. Therefore we think it needs more comprehensive studies.

Peritubal adhesions prevent the contrast agent from flowing freely around the intestines as seen in normal conditions in HSG and commonly manifests as the placement of the contrast agent around the ampullary portion of the tube^16^. In the study by Ngowa and colleagues, HSG was found to have a sensitivity of 24.6% and specificity of 45.4% in the diagnosis of pelvic adhesions^14^. In our study, 66% of the patients whose L/S indicated pelvic adhesion were found to have a bilateral or unilateral tubal obstruction in their HSG results; accordingly, we reasoned that HSG could indirectly detect pelvic adhesion in patients whose HSG results indicate a bilateral or unilateral tubal obstruction. This rate was higher than that reported in the cited study. The cited study only stated whether or not the HSG results detected pelvic adhesion and did not specify which findings were considered in the identification of pelvic adhesion. Meanwhile, we noted whether there was a tubal obstruction on HSG, and computed this rate in relation to the number of patients who demonstrated pelvic adhesion on L/S. In a L/S study conducted by Bonneau on unexplained infertile women, 83.4% of the patients with normal HSG results were detected to have pathologies^17^. We determined in our study that 37.4% of the patients with patent tubes on HSG had pathologies on L/S.

We didn't find our results compatible with this study. The number of patients in our study is more than this study. So we think that more studies are needed for the reliability of the results.

In our study, patients with L/S results indicating pathologies were found to have menstrual irregularity in their menstrual history and they were significantly different from patients who showed no pathologies on L/S in this regard (p<0.05). Therefore, we concluded that it was possible to detect pelvic pathologies using L/S in patients with irregular menstrual cycles. There was no such difference in patients who underwent HSG (p>0.05). According to our literature review, we think that more studies on this issue must be conducted to corroborate these findings.

When we look at the literature, we encountered a study comparing HSG with transvaginal hydrolaparoscopy (THL) in the diagnosis of tubal pathologies^18^. In this study, low-risk subfertile patients for tubal factor were included. HSG and THL were not performed on the same patient. We think that more detailed studies are needed for the less invasive THL.

L/S is an invasive procedure; it is associated with the risk of vascular, intestinal, bladder and ureteral injury, trocar hernia, and risks related to general anesthesia. It is also an expensive procedure. Our study concludes that HSG is not as successful as L/S in the detection of pathologies other than tubal obstruction. Therefore, diagnostic L/S is recommended if the information it provides could affect the treatment plan^19^. The inadequacy of HSG in the detection of tubal obstruction results in a need for L/S.

The limitation of our study is that it is not a prospective study but a retrospective study. Accessing patient information over the file is another limitation of our study. The fact that the number of patients is large enough to increase the reliability of the results shows the superior aspect of our study. Another strength of our study is that we perform both HSG and L / S on the same patient who is indicated.

HSG and L/S are complementary in the evaluation of tubal infertility.

While HSG is reliable when its results indicate patent tubes, L/S contributes to the identification of tubal obstructions reported by HSG and aids in the diagnosis of pelvic adhesion and other pelvic pathologies. In conclusion, patients suspected for tubal infertility can be preferred primarily undergo HSG instead of L/S, which is invasive and has higher complication rates. L/S must be considered where deemed necessary based on HSG results.
